# Considering methodological options for reviews of theory: illustrated by a review of theories linking income and health

**DOI:** 10.1186/2046-4053-3-114

**Published:** 2014-10-13

**Authors:** Mhairi Campbell, Matt Egan, Theo Lorenc, Lyndal Bond, Frank Popham, Candida Fenton, Michaela Benzeval

**Affiliations:** 1 MRC/CSO Social and Public Health Sciences Unit, University of Glasgow, Glasgow, UK; 2 NHS NIHR School of Public Health Research, London School of Hygiene & Tropical Medicine, London, UK; 3 STEaPP, University College London, London, UK; 4 Centre of Excellence in Intervention and Prevention Science, Melbourne, Australia; 5 Institute for Social and Economic Research, University of Essex, Colchester, UK

**Keywords:** Review, Theory, Systematic review

## Abstract

**Background:**

Review of theory is an area of growing methodological advancement. Theoretical reviews are particularly useful where the literature is complex, multi-discipline, or contested. It has been suggested that adopting methods from systematic reviews may help address these challenges. However, the methodological approaches to reviews of theory, including the degree to which systematic review methods can be incorporated, have received little discussion in the literature. We recently employed systematic review methods in a review of theories about the causal relationship between income and health.

**Methods:**

This article discusses some of the methodological issues we considered in developing the review and offers lessons learnt from our experiences. It examines the stages of a systematic review in relation to how they could be adapted for a review of theory. The issues arising and the approaches taken in the review of theories in income and health are considered, drawing on the approaches of other reviews of theory.

**Results:**

Different approaches to searching were required, including electronic and manual searches, and electronic citation tracking to follow the development of theories. Determining inclusion criteria was an iterative process to ensure that inclusion criteria were specific enough to make the review practical and focused, but not so narrow that key literature was excluded. Involving subject specialists was valuable in the literature searches to ensure principal papers were identified and during the inductive approaches used in synthesis of theories to provide detailed understanding of how theories related to another. Reviews of theory are likely to involve iterations and inductive processes throughout, and some of the concepts and techniques that have been developed for qualitative evidence synthesis can be usefully translated to theoretical reviews of this kind.

**Conclusions:**

It may be useful at the outset of a review of theory to consider whether the key aim of the review is to scope out theories relating to a particular issue; to conduct in-depth analysis of key theoretical works with the aim of developing new, overarching theories and interpretations; or to combine both these processes in the review. This can help decide the most appropriate methodological approach to take at particular stages of the review.

## Background

Theory is fundamental to research and rational thought. The term ‘theory’ has been variously defined, and is frequently used without definition, but often refers to an explanatory framework for observations. In science, theories generally purport to explain empirical observations and form the basis on which testable hypotheses are generated to provide support for, or challenge, the theory. Gorelick defines theory as ‘the creative, inductive, and synthetic discipline of forming hypotheses’ [1], p. 7. Popper defined a scientific theory as one that is experimentally falsifiable [2]. Merton has contrasted ‘grand’ social theories such as Marxism, functionalism, and post-modernism with ‘middle-range theories’ that start with an empirical phenomenon and abstract from it to create general statements that can be verified by data [3]. Mid-range theories are dominant within empirical and scientific approaches to research. Gough usefully categorises such research as aiming to generate, explore, or test theories. Of particular importance in health literature are studies which include theories about cause and effect; such studies may test these theories in a ‘black box’ way or attempt to generate, explore, and test more clearly articulated causal-pathway frameworks, such as those presented in logic models [4]. For this discussion, the terms ‘causal pathway’, ‘causal maps’, and ‘logic model’ refer to qualitative models used to identify key concepts and the links between them [5].

Within the health sciences, it is widely understood that individual and population health are influenced by a wide array of interconnecting factors, so theoretical models can be complex and, at times, contested [6]. However, different disciplines approach such research in different ways and are not always well connected. Reviews of theory may aid our attempts to navigate a diverse literature and potentially lead to insights into how factors relate to one another [6-9]. Theory reviews could have one or more of the following aims: identifying and mapping a comprehensive range of relevant theories; assessing which theories have become influential and which have been, or have become over time, largely overlooked; and integrating complementary theories and facilitating the analysis and synthesis of theories into more generalised or abstract ‘meta-theories’. By focusing on theory, rather than diverse empirical studies, reviews can be useful devices to describe complex topics across different disciplines and inform policy debates.

The purpose of this article is to consider the ways in which theoretical reviews might be conducted and in particular the role of systematic approaches within this. It illustrates the discussion by drawing on the approach of a recent theoretical review the authors undertook of income and health [10]. It discusses some of the methodological challenges and options that reviewers may face when planning and conducting reviews that focus on theoretical literature. We think the discussion will be particularly relevant to reviewers considering the degree to which they might attempt to use and adapt methods commonly associated with systematic reviews, which tend to have been developed around reviews of empirical research and thus not specifically designed to assess descriptions of theories underpinning research. We will discuss the extent to which methods developed and used for reviews of empirical research may, or indeed may not, be usefully adapted to meet the challenges posed when reviewing theories on the phenomena of interest. In particular, we will discuss some of the methods we (the authors) employed when conducting our own recent review of theories of income and health [10]. Reviews of theory are part of a growing methodological advancement, and we think this would be an opportune time to contribute lessons learnt from our project and others and discuss some of the methodological considerations that inform such a review. Some of our reflections are based on the methods we employed in our review; others result from critical thinking and discussions that took place following the review’s completion.

Below, we outline general approaches in the literature to conducting reviews of theories. We then describe the broad principles of our approach before providing a detailed summary of each stage of the review and the way in which we incorporated systematic approaches into them. We examine how this contributed to our understanding of the literature on income and health and reflect on the value of this approach.

### Existing approaches to reviews of theory

There is often substantial variation in the methodologies of reviews that consider theory. Some take the form of traditional literature reviews, often reliant on expert knowledge in the relevant field. Such expert knowledge allows in-depth understanding of theories and links between them. However, it can be limited to the disciplinary perspective of the reviewer, not necessarily identifying less popular or emerging theories, and cannot provide a sense of the extent to which different theories are employed in the literature. Given these limitations, some reviewers of theory have employed methodologies associated with systematic reviews such as comprehensive searches and clear criteria for including, appraising, and synthesising the literature to provide a more comprehensive picture [11,12]. Reviews of theory are thus rather different to reviews of empirical data. In particular, the primary goal of using systematic methods in the latter case is to minimise bias. In theory reviews—where it is not even clear that the concept of ‘bias’ is substantively meaningful—their main contribution may be more in ‘opening up’ reviewers’ thinking about the research topic and widening the potential space of hypothesis generation.

Often reviews of theory are conducted to assist reviewers involved in carrying out systematic reviews of intervention effectiveness. Realist review [13,14] is currently a key area of methodological development around the integration of theory into reviews of interventions. Realist reviews aim to draw out and test ‘programme theories’ about the causal pathways through which interventions work, in order to bring together evidence on effectiveness with data on implementation and context. In some cases, theory reviews may have relatively narrow inclusion criteria tied to theories about a specific intervention. However, narrow criteria do not necessarily lead to small-scale review. Two systematic reviews of theory conducted in relation to larger reviews are Baxter and Allmark’s [12] review of chest pain and medical assistance and Bonell et al.’s [11] review of theories on school environment and health: the former has a narrower research question with a search result of around 100 papers, but the latter entailed screening more than 62,000 papers.

A recent systematic review of interventions on crime, fear of crime, health, and the environment was preceded by a mapping broad review of theories that attempted to explain associations between these factors [5]. The crime review used a pragmatic approach to searching and selecting literature and did not attempt to provide a comprehensive systematic review of all theories related to the topic. Rather, the theory review aimed to construct a coherent framework for integrating relevant theories, in order to contextualise and better understand the empirical data.

### The income and health review of theory

We conducted a review of theories about causal relationships between income and health (see Additional file 1 for brief description). Given the wide-ranging literatures across disciplines, and the contested nature of many debates, we felt that a systematic approach to the review would help shed light on the range of casual paths that had been posited. Our intention was to gain some of the benefits of applying systematic review methods to a review of theory, such as clarity, comprehensiveness, and transparency. By making the literature search as systematic and transparent as possible, a review can extend beyond researcher knowledge and disciplinary background [15]. Developing inclusion criteria and devising methods to uniformly capture data across included papers strengthens objectivity [15]. By the time the included papers have been assessed, it is hoped that the explicit methods used reduce subjectivity. Once the theories are gathered through the systematic searching, screening, and extracting, the interpretation of their content at the synthesis stage may be still be at risk of subjectivity.

Reviews of theory may be particularly valuable in seeking to develop a synoptic understanding of questions where a number of different disciplines overlap. In our recent review of theories describing pathways linking individual and family income to health [10], we included theories from public health, psychology, social policy, sociology, and economics, all of which have distinct traditions and vocabularies. In addition, many of the causal pathways between income and health described by these theories are long and complex. In cases such as these, syntheses of theory can help to produce new insights about complex fields by drawing together different paradigms and translating concepts between disciplines.

The techniques developed in the crime theory review were adapted by the authors of the present article for a review of theories linking income to health across the lifecourse [10]. In the income and health review, an attempt was made to incorporate more techniques from systematic reviews, including *a priori* inclusion criteria, comprehensive electronic searching, and standardised data extraction. These methods were employed to capture theories from literature in disciplines with which we may have been less familiar. The methods used for our review are indebted to those developed by realist reviewers. However, our review focused less specifically on evaluating mid-range theories of the mechanisms and contexts of interventions and more on mapping and synthesising the whole landscape of theories around income, health, and the lifecourse. The resulting review was a methodological hybrid including elements of the earlier crime review, drawing on seminal literature to create a framework, and more standard systematic reviews. Below, we outline key review stages, illustrated by the methods used in the income and health review of theory. Challenges faced and tactics to address, these are described. The aim was to generate guidance and discussion of methods that may be useful when planning and conducting a review of theory.

## Methods

This article discusses some of the methodological issues we considered in developing the review and offers lessons learnt from our experiences. It examines the stages of a systematic review in relation to how they could be adapted for a review of theory. The issues arising and the approaches taken in the review of theories in income and health are considered, drawing on the approaches of other reviews of theory.

## Results and discussion

### Developing the research question

An explicitly stated research question is a characteristic of systematic reviews that can be adopted for reviews of theory. The question should be designed following consideration of what the end users will find useful, so consultations with potential end users may be part of the process [15]. As stated above, review questions can be broad or narrow in scope. A broad question may reflect the reviewers’ aim to scope out and map a wide range of theories within a subject area. The purpose of this income and health review was to use theory as a tool to support a larger programme of work exploring the importance of income and other aspects of family resources in determining a wide range of health and social outcomes. In contrast, if the aim is to identify theories relating to a specific phenomenon or intervention, then a narrow question may be more appropriate. For example, the review of theories of behaviour change in limiting gestational weight gain [16]; and in another review, Sherman et al. [17] used self regulatory theory to examine psychological adjustment among male partners in response to women’s cancer. Both these reviews of theory have a narrow topic, distinct from the broad scope of the income and health review.

### Assembling the team

Guidance for conducting a systematic review recommends gathering a team that includes an experienced reviewer, a subject specialist, and an information scientist with advanced knowledge of bibliographic search strategies [15], p. 85; [18]. For theory reviews, the role of the subject specialists and the stages at which their contribution is valuable may become particularly crucial. Besides helping to ensure that key papers within the field are identified for inclusion in the review, specialists can provide a detailed understanding of how different theories came to be developed, how one theory relates to another, and where the points of controversy lie. It may be useful to have input from more than one specialist if the scope of the review is multi-disciplinary or covers a subject area that is divided by rival theoretical ‘camps’. Conducting the income and health review was aided by the team including members with experience of systematic reviews, theory reviews, and information science, as well as reviewers with backgrounds in lifecourse epidemiology, social policy, and economics. We also worked with an advisory group that included end users and researchers with a range of experience in social science research.

The degree of specialist input required will be influenced by the depth of analysis required. Reviews that aim to provide in-depth synthesis, including attempts to develop meta-theories, are likely to require greater specialist input than reviews that aim to scope the various theories in the literature. This echoes guidance for other types of review. For example, Cochrane’s Qualitative and Implementation Methods group states that greater subject expertise is required to appraise the theoretical contributions made by qualitative research compared to that required simply to include or exclude relevant evidence [19]. Hence, the most appropriate team for any particular review will depend not only on the subject matter being reviewed, but also on the degree to which the review is intended primarily to be a scoping exercise or a more specialist theoretical analysis.

### Inclusion and exclusion

*A priori* inclusion and exclusion criteria are a mainstay of systematic reviews, helping to guide the literature search and ensure clear focus and transparency in the selection of studies. Frameworks for developing such criteria have been developed. For example, Cochrane advocates the use of criteria that specifies population, intervention, comparison, outcome, and study (PICOS) design. Qualitative reviewers have an alternative framework: sample, phenomenon of interest, design, evaluation, and research type (SPIDER) [20]. No comparable frameworks currently exist for theory reviews. When conducting reviews of theory, the task of creating inclusion/exclusion criteria can present challenges. First, the term ‘theory’ needs to be defined with enough precision to enable reviewers to consistently filter out papers that are insufficiently theoretical. Generic definitions, such as those highlighted at the beginning of this article, are conceptually helpful, but in practice, reviewers may find themselves struggling to decide whether a text is describing a theory or a hypothesis or speculation (and wondering how crucial these distinctions might be for the review). They may also struggle to find a consistent way of distinguishing a general discussion of issues from a more fully expounded theory. The decisions that were taken of includable theory during the income and health review were guided by screening for substantial hypothesis of exposure—mechanism—outcome and study design. We found considering papers in relation to these criteria a useful tool to clarify the theory content of papers, although we would emphasise that in this particular example it is theories of cause and effect that is being examined. Reviews of alternative types of theory would require alternative criteria.

A second challenge relates to the tension between having inclusion criteria that are specific enough to make the review practical and focused, but not so specific that key literature is excluded. Although this issue is not exclusive to reviews of theory, we found that a particular problem with the income and health theory review was that some of the most widely recognised theoretical texts did not originate from literature that focused on income, health, or lifecourse (i.e. not all three of these elements simultaneously) specifically. The Black Report [21], for instance, continues to exert a huge influence on how researchers think about the causes of health inequalities, and its theoretical framework can be seen to underpin some of the literature that was relevant to our review. However, the Black Report itself generally refers to the broader concept of socio-economic status rather than more specifically to the issue of income. We did not want to exclude the theoretical framework outlined in the Black Report nor did we want to open up our inclusion criteria so that it included all theories of socio-economic status and health (to do so would have been an enormous undertaking).

One potential solution to both these challenges is to acknowledge that overly rigid *a priori* inclusion criteria may be less useful for theory reviews, whilst more subjective methods of selecting relevant studies may be more useful; what may be required is a careful balance between these approaches. In the income and health review we developed inclusion criteria in advance of the review but acknowledged that there could be some papers key to explaining relevant theories that might be missed and therefore we allowed reviewers some leeway to include papers that did not quite fit the criteria if deemed sufficiently relevant. Subjective appraisals for relevance should ideally be conducted independently by more than one reviewer reading each text and, if necessary, resolving disagreements through discussion and/or an additional reviewer’s input. However, this process can be time consuming and resource intensive if a literature search has identified large numbers of potentially relevant papers. Another solution could be to use a second reviewer to independently check a random sample of included papers to verify that the criteria are being met consistently. Reviewers may also choose to modify inclusion criteria as the review progresses so that apparent gaps can be redressed and points of interest can be pursued in more detail [18]. Whilst potentially useful, these ‘solutions’ all carry possible risks of their own related to subjective bias, transparency, size, scope, and manageability of the review.

Our own ‘solution’ to the challenge of determining inclusion criteria was a pragmatic combination of approaches. Two of the review authors (MB, FP) with expert knowledge of socio-economics and income-health literature were able to identify ‘seminal’ papers (key texts, widely regarded as theoretically influential within the field) which are often cited as making important advances in the understanding of how socio-economic factors and health are related. The number of papers included at that stage was small, but the inclusion criteria were broad in the sense that we included theories that considered socio-economic status broadly rather than the narrow definition of income alone. From this review, we created a conceptual framework within which to structure a more in-depth review. A second stage of the review sought out wide-ranging literatures from different disciplines with theories that related specifically to income, health, and lifecourse (or life stages). For this second stage, we developed *a priori* criteria which were modified slightly as the review progressed. In addition for this stage, we developed criteria for identifying theory based broadly on Pawson and Tilley’s [22] concepts of context, mechanism, and outcome; although as noted above, the aim of our review was broader than most realist reviews and not focused on evaluation. To be included, a theory had to describe a causal association connecting income to health through a specific pathway or mechanism. More complex theories (e.g. those that involve multiple and multi-staged pathways and outcomes, feedback loops, contextual factors) were included if they involved the three core components of income, causal pathway or mechanism, and health outcome. Papers were excluded if they did not discuss theories at all or if they did not present theories containing all three of the core components. Papers were also excluded if the theoretical discussion was judged (subjectively) by reviewers to be cursory, for example, where a hypothesis or existing theory was briefly referred to or implied as part of a general discussion.

### Searching

Literature searches for systematic reviews often incorporate formal electronic searches of subject-relevant research databases such as MEDLINE, EconLit, and PsycINFO. It is also good practice to include so-called ‘hand searching’ (a misnomer, as much of this searching is also electronic) techniques. Hand searching may include expert consultations, trawls through specific journals, checking the references of included studies, and seeing where included studies have themselves been cited (some databases such as Web of Knowledge and Scopus allow for this type of forward citation tracking) [15], p. 104.

**Figure 1:**
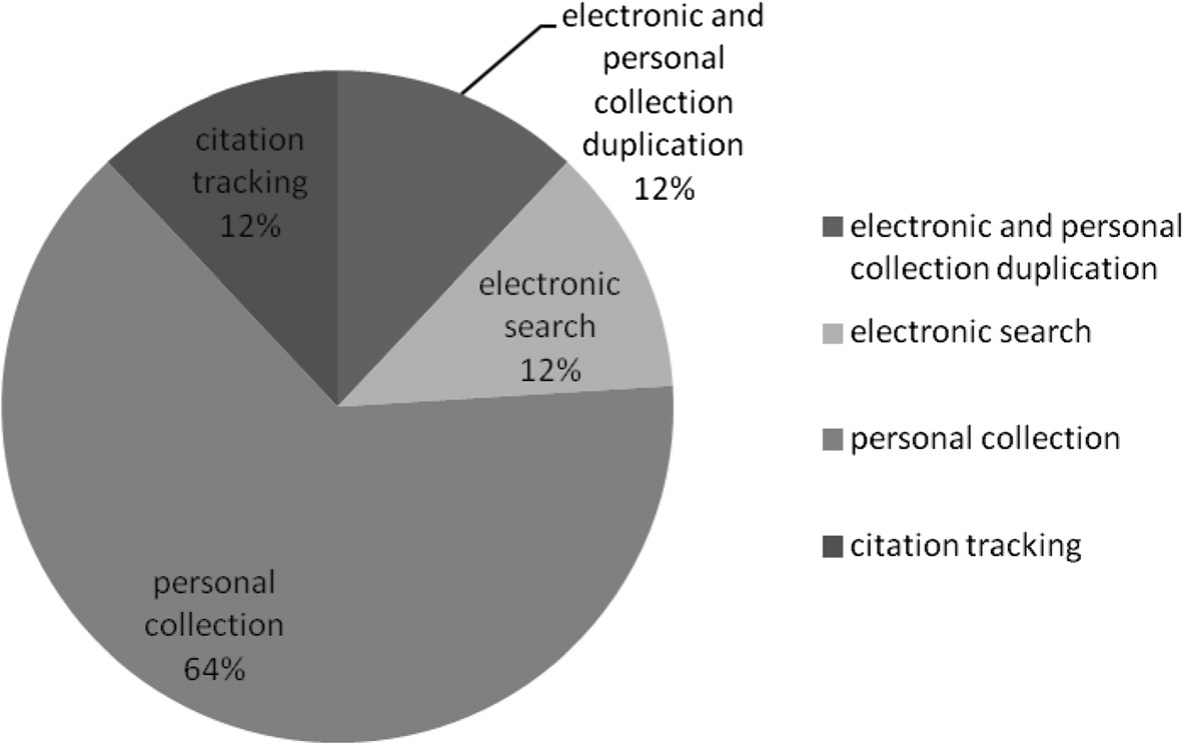
Source of included papers for the income and health review of theory.

In our review of income and health, the aim of the electronic searches was to identify papers outwith our personal collections. The intentionally broad focus of the review question, combined with the vast amount of literature relating to income and health, resulted in our development of a two part electronic search strategy (see Additional file 2). One focused on ‘highly cited literature’ and the other aimed to capture ‘recent literature’, although both employed the same search terms. The highly cited search was an attempt to identify the most influential theoretical work. This used electronic databases, SCOPUS and Web of Knowledge, which focus on high impact journals and may be used for citation tracking. The top 2,000 papers, ordered by number of citations, were taken from each database, on the assumption that the most highly cited papers were most likely to have been particularly influential. This search was repeated twice as we refined our search terms. Given the focus on highly cited papers, these searches tended to identify older papers. The ‘recent literature’ search was designed to be more specifically focused on identifying emerging theories from different disciplines. It focused on subject-specific databases from the fields of health sciences (such as epidemiology, medical sociology, health economics, health psychology, health geography, clinical sciences, public health), economics, political sciences, geography, and sociology. This search was limited to papers published within the past 10 years to identify more recent theories and those that have current application.There are no well-tested search strategies for identifying theoretical literature. Our approach identified 5,021 papers; of these, 272 were employed in the review. Several of the authors had extensive collections of papers relevant to this study, referred to here as ‘personal collection’. To satisfy our curiosity of the final contribution of the electronic and hand searching to the income and health review, we compared original search results prior to de-duplication. For this exercise, we included personal collections and citation tracking as hand searching and the ‘highly cited’ and ‘recent literature’ electronic searches as electronic. The final proportions of these groups are shown in Figure 1. Of the papers finally included in the income and health review, 76% were identified solely through hand searching: of these, 64% were in the personal collections of subject specialists and 12% came from either forward or backwards citation tracking. The citation searching included tracking references from papers found through the electronic searches. The electronic bibliographic searches identified 12% of final inclusion papers (along with a further 12% of included papers that were found both through the electronic and hand-searching methods). Therefore, for the income and health review, we found that electronic bibliographic literature searches had limitations, with a large amount of effort yielding a relatively small proportion of the final included papers. However, those papers were not identified by any of the other search strategies and hence were important to the aim of including multi-discipline literature. Citation tracking, both backwards and forwards, also resulted in useful literature being found, particularly for compiling our theoretical framework of how mechanisms interact to impact on health.

Whilst piloting our electronic search strategy, we developed and tested search terms to help us identify theoretical papers. We found that the string of terms ‘theory or pathway or model or mechanism or review’ (with truncations appropriate to specific databases) were useful for identifying papers that discussed theories. Nonetheless, when the terms for ‘income’, ‘theory’, and ‘health’ were checked separately and then combined, we also found that 17% of papers that we wanted to retrieve from our initial (and intentionally broad) electronic search did not include all of the terms in the title or abstract—and therefore would have been missed from any literature search that used that term as a filter; 8% had no term to identify theory in the title or abstract.

In other reviews, the searching process for relevant theory has been dependent on the search strategy for empirical evidence on the same topic. The theory-based review by Baxter and Allmark on chest pain and medical assistance was conducted on literature identified from a previous systematic review of empirical evidence. Hence, the reviewers focused on literature already searched and filtered [12]. The review of theory on school environment and health combined searching for theory with the searches for relevant empirical studies [23]. Therefore, papers containing relevant theory were identified during the screening process of papers reporting empirical studies. A potential limitation of this approach is that it could omit publications that provided detailed theoretical discussions without presenting empirical data. Optimising the balance between search specificity and selectivity is a perennial problem for systematic reviewers. The challenges described here underline the need for multiple approaches, including formal electronic searches and hand searches, so that the strengths of one approach can help to compensate for the deficiencies of another whilst ensuring the reviewers’ task is manageable. A consequence of the initial broad electronic searching for the income and health review was the time it took to screen over 5,000 papers. This was amplified by the fact that frequently the title and abstract gave no indication of whether there was any theoretical content and the full text had to be retrieved and screened. This is likely to be a feature of many reviews of theory and perhaps consideration has to be given to the following: achieving a balance between including search terms to limit the focus to papers including theory, with the risk of missing important texts; acknowledging a realistic timescale required for thorough searching and screening for relevant papers, with the possibility of a low ‘hit’ rate; or reconsider the objectives to establish whether a tighter focus is preferable.

### Data extraction

Reviewers have a number of options regarding how they select and extract data from included papers in such a way as to manage often substantial amounts of information and to aid synthesis. One option is to produce standardised extraction forms to help ensure that similar types of data are taken from each paper to facilitate cross-comparison. If the included documents are too heterogeneous to fit a standardised approach, or if the reviewers are looking to conduct more detailed qualitative analysis, an alternative approach is more useful. In systematic reviews of qualitative research, reviewers may work with whole texts rather than selected extracts, using ethnographic or other techniques to code and then analyse the data. Textual analysis software such as NVivo can be used to aid this process. If the reviewers feel they have a thorough knowledge of the papers, they may feel that formal data extraction and coding are unnecessary.

Reviewers of theory have a similar range of standardised and qualitative approaches to extracting data, and their choice may be determined on the purpose of the review (e.g. the extent of scoping or in-depth analysis) and their degree of familiarity with the material. As the income and health review combined an in-depth analysis of seminal literature with a broader scope of relevant theories, a combination of approaches was used. The analysis of the small number of key papers was conducted by subject specialists without a formal data extraction process. In contrast, the scoping part of the review led to the inclusion of 147 papers that were summarised using a data extraction form that we created in Microsoft Access specifically for the review (see Additional file 3 for fields included). The extracted data were then coded into broader categories of theory relating to causal mechanisms from the review of seminal papers. Data were extracted by one reviewer, and a second reviewer independently extracted a sample of studies; results were compared and differences discussed to develop a common consistent approach.

### Quality appraisal

For most systematic reviews, appraisals of the methodological quality of included evidence are a crucial stage that then enables reviewers to determine the strength of evidence and potential for bias relating to specific findings. Within evidence synthesis, in particular qualitative synthesis, there is discussion of whether it is appropriate to appraise the quality of studies and what form such appraisals might take [4,24]. Similarly, theoretical evidence cannot be appraised using the kinds of tools which have been developed for more conventional systematic reviews, most of which tend to focus on internal validity and study design. Some reviews have emphasised theories identified in empirical papers that were judged to be of high methodological quality [12]. However, study methodology and theoretical development are different areas of research demanding different skills and so it does not necessarily follow that high quality empirical methods necessarily occur alongside good or influential theories [24]. It may be that the appraisal process helps to distinguish between papers presenting a theory based on flawed empirical study and papers presenting a comprehensively argued theory which fail to clearly report research methods.

Detailed appraisal of theories is likely to involve an inductive and subjective approach by researchers with a thorough knowledge of the field, rather than the use of standardised checklists (although one exception to this is the checklist devised by Bonell et al. [23]). Our income and health review did not include a standardised critical appraisal of the theories we included. In retrospect, it may have been useful to have attempted to grade theories by relevance to the review question and, possibly, by level of detail or originality, to help exclude studies that included relatively minor theoretical discussions or simply referred to the work of other theorists.

### Synthesis

Approaches to data synthesis will differ depending on the different aims of a review. Gough helpfully distinguishes between aggregative and configurative reviews—the former generally focus on synthesising empirical papers and ‘add up’ their findings, whilst the latter aim to interpret and configure findings from existing literature to develop new understandings of existing research [4]. Theoretical reviews often lean more to configurative approaches but may also contain some aspects of aggregation depending on the aim. This results in different approaches to synthesis. Some reviews (e.g. Bonell et al. [11]) have treated the individual theory as the unit of analysis, with a focus on constructing a typology of theories within an overarching picture of causal determinants. Others have an in-depth or configurative approach, for example, Lorenc et al. [25] aimed to analytically isolate specific causal or interpretive assertions from diverse theories and then to develop a causal ‘map’ of the interrelations between different factors.

However, there remain a number of unanswered questions around synthesis of theories, particularly whether diverse, complex, and potentially incommensurable conceptual vocabularies can be effectively integrated. A review of theory that attempted a more in-depth analysis could incorporate techniques developed for qualitative reviews, for example, from thematic synthesis [26] or meta-ethnography [27]. The reviewers could attempt to distinguish between different orders of theory: those framed directly around specific data, those that result from an author’s attempts to juxtapose pre-existing theories and/or ideological positions with empirical observations, and those resulting from the reviewers own reflections based on comparisons of the included literature. Broadly following the meta-ethnographical approach, reviewers could explore whether the relationship between different theories is reciprocal (i.e. the theories are mutually supportive) or refutational (i.e. the theories appear to contradict one another) or whether the theories can potentially form part of the same line of argument (e.g. by representing different stages along the same causal pathway) [27].

As has been noted, the income and health review was a hybrid that combined an expert review of key literature with a wider scope of relevant theoretical literature drawn from systematic searches. The seminal texts in the expert review were synthesised through a subjective process of induction by specialists who had immersed themselves in the literature. A key synthesising stage of the systematic searches was the interpretative collating of findings which was used to create a causal map and review the key concepts and relations that were believed to be important. Through an iterative process of checking between this mapping process and the themes we had developed from the systematic scoping literature, a framework of theoretical pathways between income and health was constructed.

The synthesis process used in the income and health review combined standardised and iterative elements. Guided by Baxter et al.’s [6] method of developing a conceptual framework, papers were scanned to extract descriptions of specific pathways/theories linking income to health. The extracted literature was organised by a coding framework: a typology of theories, developed iteratively in conjunction with the analysis of seminal texts. The extracted texts were then organised by themes emerging from the data within each theory type, drawing together similar theoretical pathways from differing disciplines including sociology, economics, public health, and psychology. Narrative synthesis techniques were used to scope, compare, and contrast the key theories that were identified and focused on: the definition of key concepts, hypothesised pathways, the range of contextual factors included in the model/theory, and the time sequencing of hypothesised influences and outcomes within the lifecourse. These methods are similar to the processes involved in thematic synthesis described by Thomas and Harden [26]. In retrospect, awareness that the synthesis process we undertook would concentrate entirely on qualitative techniques would have enabled us to adopt qualitative software and analysis methods at an early stage of the review. This may have made the data collection quicker and the synthesis more intuitive.

## Conclusions

In this article, we have discussed some of the methodological issues involved when conducting a review of theory, using examples from a recently conducted theoretical review of income and health. The article should be read as a discussion of what we learnt rather than an attempt at formal guidance. The aim has been to help provide a starting point for anyone considering their own review of theory to think about the possible purpose of their review and the methods that are most appropriate for that purpose. We suggest that there are a spectra of methods for conducting theory reviews that stretch from scoping out theories relating to a particular issue to in-depth analysis of key theoretical works with the aim of developing new, overarching theories and interpretations. The two types of approach are not mutually exclusive; the income and health review included elements of both. We think it may be useful at the outset of a review of theory to spend time considering whether the key aim of the review is to scope, to conduct in-depth analysis, or to combine both these aspects in the review. Identifying the type of review can clarify the most appropriate approach. Scoping reviews are more likely to require a more standardised approach to searching, inclusion and exclusion, and data extraction to help manage the potentially large numbers of studies that may be identified. However, in our experience, scoping reviews of theory also benefit from the flexibility and nuance that can come from more subjective and inductive processes. In-depth reviews of theory are likely to involve iterations and inductive processes throughout, and we have suggested that some of the concepts and techniques that have been developed for qualitative evidence synthesis can be usefully translated to theory reviews of this kind.

Reflecting on our experience of conducting the review of theories on income and health, we feel that there were positive and negative aspects to the process. Table 1 summarises the main challenges we faced. Taking the time to grapple with defining and applying inclusion criteria was a process which helped clarify what we were looking for and how we wanted to use it. The systematic searching was extensive and laborious, and we found that it contributed only a small amount to the review in comparison with that found through personal libraries. However, those papers would have been omitted without the systematic search methods, probably reducing the scope of the review.

**Table 1:** Challenges of applying systematic review methods to a review of theory

**Review stage**	**Systematic review**	**Challenge for income-health review**	**Our resolution**
Inclusion criteria	Emphasis on setting *a priori* criteria specifying population, intervention, comparison, outcome, and study (PICOS) design; or SPIDER [20] for reviews of qualitative data.	Defining a theory. Subject-specific inclusion criteria may exclude relevant theories that originated in different subject areas.	To be included, a theory had to define a mechanism linking financial resources and health. Subject experts identified influential theories that originated from other subject areas.
Literature search	Generally strong focus on searches of electronic bibliographic databases, requiring clear search terms.	Vast literature and intentionally broad review question including literature from different subject areas, using different terminologies.	Review included multiple forms of both formal electronic searches and hand searches and citation tracking to follow how theories develop and influence later literature. This is less of an issue if the search relates to a narrower review question, e.g. focusing on a specific intervention and its mechanism.
Data extraction	Standardised forms and software (e.g. RevMan) available	Determining what information is required to extract and how best to extract this uniformly. Various methods possible.	Developed a spreadsheet for extracting data about theories from a large number of included studies. This process could have been improved by using qualitative methods and software.
Quality appraisal	Appraisal of clear reporting of empirical study methods to enable assessment of potential biases, and the generalisability of the study results.	Tools developed for appraising conventional systematic reviews focus on internal validity and study design.	The theory review did not include a standardised critical appraisal of the theories. In retrospect, it may have been useful to have attempted to grade theories by relevance to the review question and, possibly, by level of detail or originality [23].
Synthesis	Various methods, including meta-analysis of suitably homogeneous data and narrative synthesis to explore heterogeneity between studies.	Summarising theories rather than empirical findings.	Interpretative collation of key concepts and relations to create a causal map. Iterative process of checking between this mapping process and the themes emerging from the data. Consideration of how competing theories may be genuinely oppositional, mutually inclusive, and/or represent different links in a longer and more complex causal chain (similar to meta-ethnographic concepts of reciprocity, refutation, and line of argument).

Our team of authors included members with substantial knowledge of the income and health topic; it is possible that conducting the review without access to this knowledge would make the systematic searching of far greater relevance. The team also included members with considerable experience in conducting systematic reviews. Although this had some advantages in terms of methodological expertise, we have tried to show here that systematic review methods are not always appropriate or may need to be adapted for theory reviews—and the reviewers need the confidence and flexibility to do this.

We suggested in the background above that systematic methods may be valuable in reviews of theory for two reasons: to complement the review team’s existing expertise as a framework for hypothesis generation and to increase reliability in the synthesis. Our experience suggests that these benefits are real, in that the systematic approach helps to distance reviewers from commitments to particular perspectives. Nonetheless, reviewer expertise continued to define the interpretation of the theories and was also indispensable in searching.

Reviewers wondering which approach and methods would best suit them should consider the purpose of the review in terms of what would be most useful to end users. Furthermore, if reviews of theory are to be more common, their general utility requires greater consideration. At this stage in their development, there is an opportunity to pose searching questions about the uses and usefulness of such reviews. How would the focus and content alter depending on whether their main function was as an academic resource, as a support for decision makers, or as a combination of the two? Do their findings genuinely advance our understanding of theory (e.g. by identifying overlooked theories, by showing how apparently rival theories relate to one another, or by aiding the generation of meta-theories)? Conversely, do they tend to reiterate theoretical viewpoints that are already well established (including, for example, the view that social phenomena are frequently ‘complex’)? Future work on this kind of synthesis will doubtless lead to a refinement of methods and can shed more light on the added value that can be obtained by reviewing theory.

## Competing interests

The authors declare that they have no competing interests.

## Authors’ contributions

MC and ME wrote the first draft of this article, with MB and TL contributing to drafting. All authors contributed to revising the manuscript and approved the final draft. All authors were involved in developing and conducting the income health review of theory.

## Supplementary Material

Additional file 1 **Purpose and methods for the income and health review [**[21]**,**[28]**-**[30]**].** The review is on theories about causal relationships between income and health. Click here for file

Additional file 2 **Further details of income and health electronic literature searches.** Details show development of a two part electronic search strategy. Click here for file

Additional file 3 **Data extraction for the income and health review [**[30]**-**[41]**].** List shows the details collected from the papers included in the systematic search. Click here for file

## References

[B1] Gorelick R What is theory? Ideas EcolEvol 2011 4 1 10

[B2] Popper K The Logic of Scientific Discovery 1959 London: Routledge

[B3] Merton RK Social Theory and Social Structure 1968 New York: Free Press

[B4] Gough D Thomas J Oliver S Clarifying differences between review designs and methods Syst Rev 2012 1 28 10.1186/2046-4053-1-28 22681772 PMC3533815

[B5] Lorenc T Petticrew M Whitehead M Neary D Clayton S Wright K Thomson H Cummins S Sowden A Renton A Crime, fear of crime and mental health: synthesis of theory and systematic reviews of interventions and qualitative evidence Public Health Res 2013 13 496 10.1186/1471-2458-13-496 PMC3666893 23705936

[B6] Baxter S Killoran A Kelly MP Goyder E Synthesizing diverse evidence: the use of primary qualitative data analysis methods and logic models in public health reviews Public Health 2010 124 99 106 10.1016/j.puhe.2010.01.002 20167340

[B7] Tugwell P Petticrew M Kristjansson E Welch V Ueffing E Waters E Bonnefoy J Morgan A Doohan E Kelly MP Assessing equity in systematic reviews: realising the recommendations of the Commission on Social Determinants of Health BMJ 2010 341 c4739 10.1136/bmj.c4739 20837575

[B8] Anderson LM Petticrew M Rehfuess E Armstrong R Ueffing E Baker P Francis D Tugwell P Using logic models to capture complexity in systematic reviews Res Synth Methods 2011 2 33 42 10.1002/jrsm.32 26061598

[B9] Lorenc T Pearson M Jamal F Cooper C Garside R The role of systematic reviews of qualitative evidence in evaluating interventions: a case study Res Synth Methods 2012 3 1 10 10.1002/jrsm.1036 26061997

[B10] Benzeval M Bond L Campbell M Egan M Lorenc T Petticrew M Popham F How does Money Influence Health? 2014 York: Joseph Rowntree Foundation

[B11] Bonell CP Fletcher A Jamal F Wells H Harden A Murphy S Thomas J Theories of how the school environment impacts on student health: systematic review and synthesis Health Place 2013 24 242 249 24177419 10.1016/j.healthplace.2013.09.014

[B12] Baxter S Allmark P Reducing the time-lag between onset of chest pain and seeking professional medical help: a theory-based review BMC Med Res Methodol 2013 13 15 10.1186/1471-2288-13-15 23388093 PMC3570316

[B13] Pawson R Greenhalgh T Harvey G Walshe K Realist synthesis: an Introduction 2004 ESRC Research Methods Programme Manchester: University of Manchester

[B14] Pearson M Chilton R Woods HB Wyatt K Ford T Abraham C Anderson R Implementing health promotion in schools: protocol for a realist systematic review of research and experience in the United Kingdom (UK) Syst Rev 2012 1 48 10.1186/2046-4053-1-48 23083508 PMC3488465

[B15] Petticrew M Roberts H Systematic reviews in the social sciences: A Practical Guide 2006 Oxford: Blackwell

[B16] Hill B Skouteris H Fuller-Tyszkiewicz M Interventions designed to limit gestational weight gain: a systematic review of theory and meta-analysis of intervention components Obes Rev 2013 14 6 435 450 10.1111/obr.12022 23534901

[B17] Sherman KA Kasparian NA Mireskandari S Psychological adjustment among male partners in response to women’s breast/ovarian cancer risk: a theoretical review of the literature Psycho-Oncology 2010 19 1 11 10.1002/pon.1582 19472298

[B18] Bambra C Real world reviews: a beginner’s guide to undertaking systematic reviews of public health policy interventions J Epidemiol Community Health 2011 65 14 19 10.1136/jech.2009.088740 19710043

[B19] Hannes K Noyes J, Booth A, Hannes K, Harden A, Harris J, Lewin S, Lockwood C Critical appraisal of qualitative research Supplementary Guidance for Inclusion of Qualitative Research in Cochrane Systematic Reviews of Interventions 2011 The Cochrane Collaboration Qualitative and Implementation Methods Group http://cqim.cochrane.org/supplemental-handbook-guidance

[B20] Cooke A Smith D Booth A Beyond PICO: the SPIDER tool for qualitative evidence synthesis Qual Health Res 2012 22 1435 1443 10.1177/1049732312452938 22829486

[B21] Department of Health and Social Security Inequalities in Health: Report of a Working Group, Chaired by Sir Douglas Black 1980 London: DHSS

[B22] Pawson R Tilley N Realistic Evaluation 1997 London: Sage Publications Ltd

[B23] Bonell C Jamal F Harden A Wells H Parry W Fletcher A Petticrew M Thomas J Whitehead M Campbell R Murphy S Moore L Systematic review of the effects of schools and school environment interventions on health: evidence mapping and synthesis Public Health Res 2013 1 doi:10.3310/phr01010 25642578

[B24] Campbell R Pound P Morgan M Daker-White G Britten N Pill R Yardley L Pope C Donovan J Evaluating meta-ethnography: systematic analysis and synthesis of qualitative research Health Technol Assess 2011 15 43 22176717 10.3310/hta15430

[B25] Lorenc T Clayton S Neary D Whitehead M Petticrew M Thomson H Cummins S Sowden A Renton A Crime, fear of crime, environment, and mental health and wellbeing: mapping review of theories and causal pathways Health Place 2012 18 757 765 10.1016/j.healthplace.2012.04.001 22542441

[B26] Thomas J Harden A Methods for the thematic synthesis of qualitative research in systematic reviews BMC Med Res Methodol 2008 8 45 10.1186/1471-2288-8-45 18616818 PMC2478656

[B27] Noblit GW Hare RD Meta-ethnography: Synthesizing Qualitative Studies 1988 London: Sage Publications Inc

[B28] Whitehead M Townsend P, Whitehead M, Davidson N **The health divide** Inequalities in Health: the Black Report and the Health Divide. edn 1992 London: Penguin 213

[B29] Macintyre S The Black Report and beyond: what are the issues? Soc Sci Med 1997 44 6 723 745 10.1016/S0277-9536(96)00183-9 9080558

[B30] Adler NE Stewart J Health disparities across the lifespan: meaning, methods, and mechanisms Ann NY Acad Sci 2010 1186 5 23 10.1111/j.1749-6632.2009.05337.x 20201865

[B31] Kroenke C Socioeconomic status and health: youth development and neomaterialist and psychosocial mechanisms Soc Sci Med 2008 66 31 42 10.1016/j.socscimed.2007.07.018 17868964

[B32] Friedman EM Love GD Rosenkranz MA Urry HL Davidson RJ Singer BH Ryff CD Socioeconomic status predicts objective and subjective sleep quality in aging women Psychosom Med 2007 69 682 691 10.1097/PSY.0b013e31814ceada 17766692

[B33] Klabbers G Bosma H Van Lenthe FJ Kempen GI Van Eijk JT Mackenbach JP The relative contributions of hostility and depressive symptoms to the income gradient in hospital-based incidence of ischaemic heart disease: 12-year follow-up findings from the GLOBE study Soc Sci Med 2009 69 1272 1280 10.1016/j.socscimed.2009.07.031 19713020

[B34] McLoyd VC The impact of economic hardship on black families and children: psychological distress, parenting, and socioemotional development Child Dev 1990 61 311 346 10.2307/1131096 2188806

[B35] Huston AC McLoyd VC Coll CG Children and poverty: issues in contemporary research Child Dev 1994 65 275 282 10.1111/j.1467-8624.1994.tb00750.x 7516847

[B36] Pearlin LI Schieman S Fazio EM Meersman SC Stress, health, and the life course: some conceptual perspectives J Health Soc Behav 2005 46 205 219 10.1177/002214650504600206 16028458

[B37] Theodossiou I Zangelidis A The social gradient in health: the effect of absolute income and subjective social status assessment on the individual’s health in Europe Econ Hum Biol 2009 7 229 237 10.1016/j.ehb.2009.05.001 19497794

[B38] Jeffery RW French SA Socioeconomic status and weight control practices among 20- to 45-year-old women Am J Public Health 1996 86 1005 1010 10.2105/AJPH.86.7.1005 8669502 PMC1380443

[B39] Pampel FC Krueger PM Denney JT Socioeconomic disparities in health behaviors Annu Rev Sociol 2010 36 349 370 10.1146/annurev.soc.012809.102529 21909182 PMC3169799

[B40] Raphael D Macdonald J Colman R Labonte R Hayward K Torgerson R Researching income and income distribution as determinants of health in Canada: gaps between theoretical knowledge, research practice, and policy implementation Health Policy 2005 72 217 232 10.1016/j.healthpol.2004.08.001 15802156

[B41] Cerdá M Johnson-Lawrence VD Galea S Lifetime income patterns and alcohol consumption: investigating the association between long- and short-term income trajectories and drinking Soc Sci Med 2011 73 1178 1185 10.1016/j.socscimed.2011.07.025 21890256 PMC3185179

