# Supplementary material for: Considering methodological options for reviews of theory: illustrated by a review of theories linking income and health
Source: Syst Rev. 2014 Oct 13;3:114. doi: 10.1186/2046-4053-3-114 (PMC4208031; doi:10.1186/2046-4053-3-114)
Supplement: Additional file 1 — Purpose and methods for the income and health review [[21],[28]-[30]]. The review is on theories about causal relationships between income and health. [file 2046-4053-3-114-S1.doc]

Additional file 1: Purpose and methods for the income and health review

The purpose of the income and health review was to develop a theoretical framework that describes causal pathways between individual and family income and health over the lifecourse. This theoretical approach aimed to identify and make explicit assumptions that drive empirical research and its different interpretations.

Income and health research is a subset of broader literatures on socio-economic factors and health. The focus on income per se is sometimes due to substantive interest in specific mechanisms that might be important but more often due to the data available, disciplinary background of authors, or tradition of the country in which the research is being conducted. The broad theoretical framework explaining the association between socio-economic factors and health was set out in the Black Report [21] – this was based on a government enquiry commissioned by a Labour Government but received by the incoming Conservative one. The report findings were rejected and its publication was buried, leading to considerable academic hostility [28]. Subsequent decades saw polarisation of research and policy; with the dominant academic approach focusing on structural arguments while policy makers would only contemplate individualist behavior explanations. Different disciplines took very different foci (for example, economists often focused on pathways from health to income while sociologists and social epidemiologists believed it ran the other way). The literature includes a number of acrimonious debates with often very personal attacks on academics that investigated alterative explanations [29]. As longitudinal cohort studies began to inform debates, new theories emerged and began to dominate debates [30]. When a new Government took office in the late 1990s, demands for research on ‘what works to tackle inequalities’, led to new demands and kinds of research with theory having a different kind of role. All of these different drivers have led to vast literature on social inequalities in health, with distinct phases of foci and many contested theories and empirical findings. The specific place of debates about income per se within this are difficult to identify from disparate literatures that employ it as a measure for different reasons. For all these reasons we felt that a systematic review might aid understandings of the underlying theories that link income and health. The project: (1) described the broad theoretical frameworks that explain how socio-economic factors might influence health, and identified key concepts and debates on important contexts for the income and health association specifically; (2) used systematic review methods to describe how the broad theories are operationalised into specific mechanisms and pathways, illustrated with evidence from different disciplines; (3) drew the different theories together, and demonstrated their interconnectedness with a series of case studies.

The methodology comprised of two approaches. For the overall framework and concept definition, our existing knowledge and literature libraries were used to identify broad theories of how socio-economic position may influence health. Then systematic searches of literature were conducted to identify how these broad theories were articulated, employed and developed in relation to income and health, and uncover any developing theories or key theories that may have been missed from the broad framework.
