# Supplementary material for: Considering methodological options for reviews of theory: illustrated by a review of theories linking income and health
Source: Syst Rev. 2014 Oct 13;3:114. doi: 10.1186/2046-4053-3-114 (PMC4208031; doi:10.1186/2046-4053-3-114)
Supplement: Additional file 2 — Further details of income and health electronic literature searches. Details show development of a two part electronic search strategy. [file 2046-4053-3-114-S2.doc]

Additional file 2: Further details of income and health electronic literature searches

‘Highly cited’ search:

- Databases searched were Scopus and Web of Knowledge, August 2012, January 2013
- The first two iterations of this search used terms sets for ‘income’ and’ ‘health’. The third iteration added terms for ‘theory’
  - Terms to identify ‘income’: financial difficult*; income support; personal finance; public assistance; social security; disability benefit*; earning*; economic*; income; money; pension*; poverty; salaries; salary; wage*; wealth* expenditure*; spending*; living standards; standards of living.
  - Terms to identify health: life expectancy; medical condition*; quality of life; well being; death; disease*; happiness; health*; hospitalisation; illness; lifespan; malaise; morbidity; mortality; QOL; wellbeing.
  - Terms to identify theory: theory; pathway; model; mechanism; longitudinal; cohort; lifecourse; review
- Terms were combined using Boolean ‘OR’ within a set, and sets combined using Boolean ‘AND’
- Search results were then sorted by ‘most cited’
- Top 2000 citations were exported to reference management software and de-duplicated for sifting

‘Recent literature’ search:

- Databases searched were CINAHL, Econlit, Embase, IBSS , Pubmed, RePEc, and Socindex, October 2012
- Terms sets for three concepts were developed: ‘income’; health; theory (based on term above)
- Terms were combined using Boolean ‘OR’ within a set, and sets combined using Boolean ‘AND’
- Searches were restricted to published in previous ten years, English language only, and ‘developed’ countries
