# Supplementary material for: Considering methodological options for reviews of theory: illustrated by a review of theories linking income and health
Source: Syst Rev. 2014 Oct 13;3:114. doi: 10.1186/2046-4053-3-114 (PMC4208031; doi:10.1186/2046-4053-3-114)
Supplement: Additional file 3 — Data extraction for the income and health review [[30]-[41]]. List shows the details collected from the papers included in the systematic search. [file 2046-4053-3-114-S3.doc]

Additional file 3: Data extraction for the income and health review

The following details were collected from the papers included in the systematic search:

**General details of the paper**: search origin; author and date; title; type of paper (e.g. review paper, primary quantitative study) and type of study.

**Population focus**: lifestage under examination (e.g. adult, childhood); population (e.g. if focus on men); country of study.

**Exposure, mechanism, outcome details**: financial resources description; health description; outline of the mechanism/pathway

**Theories**: All theories mentioned in paper (verbatim); details of each theory discussed in the paper (the final list of theory categories comprised: artifact; selection; behavioural; material; psychosocial; lifecourse; ‘other’ theory).

If relevant, whether theory was tested and if findings supported or refuted it; any policy implications explicitly reported.

Examples of pathways developed during data extraction and synthesis:

***Low income*** *→* ***Economic deprivation*** *→* ***multiple daily stressors (****→****depression, hostility)*** *→* ***influences lifestyle, unhealthy behaviours*** *→* ***ill health*** (compiled from: [30-37]

***Income*** *→* ***social support/control at work/work–life balance*** *→* ***stress*** *→* ***health*** (compiled from: [33, 38-41]
